# Recruiting patients into a healthcare services trial: lessons learned from a feasibility study to investigate a patient-oriented navigation intervention for age-associated diseases

**DOI:** 10.1186/s12913-025-13023-x

**Published:** 2025-07-02

**Authors:** Kathrin Gödde, Hella Fügemann, Ulrike Grittner, Raphael Kohl, Andreas Meisel, Thomas Reinhold, Nina Rieckmann, Susanne Schnitzer, P. Markus Deckert, Nikolaj Frost, Christian H. Nolte, Stephan J. Schreiber, Ute Goerling, Christine Holmberg

**Affiliations:** 1 Institute of Public Health, Charité—Universitätsmedizin Berlin, Corporate member of Freie Universität Berlin and Humboldt-Universität zu Berlin, Berlin, Germany; 2 Brandenburg Medical School Theodor Fontane, Institute of Social Medicine and Epidemiology, Brandenburg an der Havel, Germany; 3 Institute of Biometry and Clinical Epidemiology, Charité—Universitätsmedizin Berlin, Corporate member of Freie Universität Berlin and Humboldt-Universität zu Berlin, Berlin, Germany; 4 Institute of Medical Sociology and Rehabilitation Science, Charité—Universitätsmedizin Berlin, Corporate member of Freie Universität Berlin and Humboldt-Universität zu Berlin, Berlin, Germany; 5 Center for Stroke Research Berlin (CSB), Department of Neurology with Experimental Neurology, Charité—Universitätsmedizin Berlin, Corporate member of Freie Universität Berlin and Humboldt-Universität zu Berlin, Berlin, Germany; 6 Institute of Social Medicine, Epidemiology and Health Economics, Charité—Universitätsmedizin Berlin, Corporate member of Freie Universität Berlin and Humboldt-Universität zu Berlin, Berlin, Germany; 7 Department of Hematology, Oncology and Palliative Care, Brandenburg Medical School Theodor Fontane, Brandenburg an der Havel, Germany; 8 Department of Infectious Diseases and Pulmonary Medicine, Campus Virchow-Klinikum and Campus Charité Mitte, Charité-Universitätsmedizin Berlin, Corporate Member of Freie Universität Berlin and Humboldt-Universität zu Berlin, Berlin, Germany; 9 Department of Neurology, Oberhavel Kliniken, Clinic Hennigsdorf, Hennigsdorf, Germany; 10 Charité Comprehensive Cancer Center, Charité—Universitätsmedizin Berlin, Corporate member of Freie Universität Berlin and Humboldt-Universität zu Berlin, Berlin, Germany; 11 Faculty of Health Sciences, Brandenburg Medical School Theodor Fontane, Neuruppin, Germany

**Keywords:** Navigation intervention, Age-associated diseases, Study recruitment, Process evaluation

## Abstract

**Background:**

Interventions to improve care coordination, like patient navigation programs, aim to dismantle barriers faced by patients in accessing optimal care. A variety of interventions are currently being evaluated in Germany and internationally. A key challenge of these studies, as for trials in general, is finding an effective recruitment strategy to reach the intended sample size in the targeted population.

**Methods:**

Detailed documentation of the recruitment process was conducted as part of the process evaluation for a mixed-methods feasibility study including randomized trials and parallel cohort studies to evaluate a patient-oriented navigation program. Patients with lung cancer and stroke were actively recruited in inpatient and specialized outpatient settings in a rural and a metropolitan area in Germany between June 2021 and September 2022. Reasons for excluding or not approaching patients were documented and patients’ reasons for refusal were assessed. All quantitative data were analysed in a descriptive manner. Experiences during the recruitment process were investigated through interviews with recruiting personnel and analysed through thematic analysis.

**Results:**

The data from the screening and recruitment process show that 74-76.5% of stroke patients and 91–93% of lung cancer patients were eligible to take part in the study. Of these, 44-46.9% of inpatients and 73% of outpatients were actively approached for recruitment. Reasons for not approaching patients were mainly due to organizational and contextual factors. Documented reasons for patients’ refusal to participate in the study included feeling overwhelmed (stroke patients) and not perceiving the study as relevant (lung cancer patients).

**Conclusions:**

The presented experiences and barriers during the recruitment process for a feasibility study of a patient navigation program provide important lessons for future planning of appropriate recruitment strategies to enrol patients with age-associated diseases.

**Trial registration:**

The study was registered at the German Clinical Trials Register (DRKS-ID: DRKS00025476, Registration Date: 04/06/2021).

**Supplementary Information:**

The online version contains supplementary material available at 10.1186/s12913-025-13023-x.

## Introduction

Finding an appropriate and successful strategy for patient recruitment is critical for study development and planning, as failure to achieve the intended case number can diminish the validity of a trial and the informative value of the effects on investigated outcomes [1, 2]. However, finding appropriate strategies to achieve target sample sizes appears to be one of the major challenges in planning and conducting a trial. A systematic review from the UK investigating recruitment success shows that of over one hundred multicenter clinical trials, less than a third achieved the intended sample size [3–5]. Factors and barriers associated with difficulties and failure to achieve planned recruitment numbers range from organizational, study-related as well as patient-related factors [6]. Systematic reviews investigating the influence of different strategies to improve recruitment have not yielded conclusive results [3]. Factors identified to improve recruitment rates include effective trial communication (e.g. face-to-face in addition to written study information, concise information that is free of medical jargon, information delivery by a person with good communication skills), an open (versus a blind) trial design, and telephone or SMS reminders after a written invitation [3, 7]. Further previous studies report selection bias and underrepresentation of patient populations due to barriers in enrolment and consent processes, for instance due to severe cognitive deficits following stroke [8, 9] or language barriers [10, 11].

A variety of questions and factors thus needs to be considered when planning the recruitment process for a healthcare intervention, such as: (a) In which (care) *setting* can the targeted study population be comprehensively reached for recruitment (e.g. at inpatient clinics, rehabilitation offers, ambulatory practices)? (b) Which *time point* along patients’ care pathways is most appropriate for reaching out to them and offering the specific care intervention that is being investigated? Notably, the time point and care setting in which patients can be most comprehensively approached are not necessarily in line with the optimal time point or care setting in which patients are most in need and hence most open to participating in an intervention study [7]. Furthermore, (c) The *mode of contact* of the recruiting persons needs to be decided upon. On the one hand, it is considered advantageous to employ in-house personnel for recruitment due to their easier access to and better knowledge of the recruitment site and to prevent data protection issues from having external recruiters approaching patients. On the other hand, integrating time-consuming recruitment activities into an already high workload at hospitals and other care facilities is difficult and can lead to work overload and low prioritization of study recruitment in routine practice [12–14]. Moreover, it is important to take into consideration and to investigate (d) *Patients’ reasons* for participating or not in intervention studies. Previous studies have shown that the perception of a potential personal benefit from the intervention or the chance to help others are factors that motivate patients to take part in studies [7]. On the other hand, the high time commitment required to participate in a study as well as the feeling of overwhelm due to patients’ individual health situation at the time of enrolment may negatively impact their willingness to participate [7, 14, 15].

For an effective recruitment strategy, all of the above factors have to be taken into consideration and balanced in a strategy that also takes into account a project’s financial and personnel resources. In this paper, we describe the recruitment process for a study to investigate the feasibility of a patient navigation intervention for patients with stroke and lung cancer. Patient navigators have the role of supporting patients with their care coordination and organization where they may face difficulties especially due to the highly fragmented German health care system. Especially patients of older age or without much social support may be in special need of navigational support [16]. To recruit participants into the CoreNAVI study, patients were actively approached in inpatient and specialized outpatient settings by the project’s study personnel. Part of the feasibility assessment was to investigate, in an accompanying process evaluation, whether the planned recruitment strategy was appropriate and efficient for reaching and enrolling the targeted patient population, or whether alternatives should be considered. The recruitment process was thus documented in detail to gain insight into who could be reached in the described care settings and why patients were not enrolled into the study (these included organizational, contextual, as well as patients’ personal reasons). Hence, the presented results aim to provide observations and experiences to give information on a common challenge: Which factors influence patient participation and refusal in studies investigating new healthcare interventions, and which should be considered when designing such studies?

## Methods

### Study design

The data presented here were part of a mixed-methods feasibility study aimed at evaluating a patient-oriented navigation program for patients with age-associated diseases (stroke and lung cancer) [17]. We conducted two-arm randomized controlled trials (RCTs) with parallel cohorts, qualitative interviews, and secondary data analyses of health insurance records. The primary aim of the underlying study was to evaluate the program’s feasibility regarding acceptance, demand and practicality, as well as to investigate first efficacy outcomes. The data presented here focus on the process evaluation that accompanied the screening and recruitment phases, with the aim of evaluating the chosen screening and recruitment strategy. The process evaluation included different methods, namely a detailed documentation of the screening and recruitment processes, a refusal assessment, as well as qualitative interviews with the recruiters. Further details on the study design of the feasibility study are described in the published study protocol, see Gödde et al. [17]. The study was registered at the German Clinical Trials Register (DRKS-ID: DRKS00025476, Registration Date: 04/06/2021).

### Recruitment strategy

Participants with either acute stroke or lung cancer and their caregivers were actively approached to take part in a feasibility study to test a patient navigation program. All potential participants were invited to take part in a two-arm RCT, where the intervention group received active support by a personal navigator for one year while the control group received a brochure with regional support offers. If those approached did not want to take part in the RCT, they were offered to take part in a parallel cohort study where patients were not randomized and did not receive a navigation intervention. In all study arms (RCT and cohort), participants underwent temporally- and content-related parallel assessments, including a baseline assessment with questionnaires at the time of enrolment, followed by three follow-up assessments conducted at four, seven, and 13 months post-enrolment. The intervention group received additional questions in follow-up questionnaires regarding their experience with the navigation intervention. The study was approved by the Ethics Committees of the Charité– Universitätsmedizin Berlin (EA2/249/20) and Medizinische Hochschule Brandenburg– Theodor Fontane (Z-01–20210517).

Inclusion criteria were diagnosis of acute stroke/TIA (transient ischemic attack) or lung cancer, aged 18 years and older, and resident in a predefined catchment area (the metropolitan area of the city of Berlin or the German federal state of rural Brandenburg). Exclusion criteria were residency in a nursing home at the time of enrolment, a language barrier (non-German-speaking), and dementia at the time of enrolment. Eligible patients were pre-screened for inclusion and exclusion criteria by the collaborating trial unit or in cooperation with the medical personnel at the participating recruitment site and were reported to the project’s recruitment staff daily.

Regarding the mode of contact for recruitment, eligible participants or their caregivers were actively approached in person by the project’s recruitment personnel (study nurses, social worker). For the recruitment sites, we chose inpatient settings (two stroke units in Berlin and one stroke unit in rural Brandenburg, lung cancer patients in Brandenburg) and a specialized outpatient clinic for lung cancer patients in Berlin. These settings were chosen with the intention of comprehensively reaching the intended target group (for a detailed description of the recruitment sites, see Table 1). Stroke patients were approached shortly after their stroke incident. Lung cancer patients were approached during their outpatient therapy phase. Potential participants were contacted in-person at the recruitment site and were provided with oral and written information on the CoreNAVI study. Recruitment mostly took place directly at the recruitment sites. If more time or consultation with caregivers was requested, a later time was agreed upon or consent for future contact by the study staff was obtained. In case of study enrolment, written informed consent was obtained. Comprehensive information on the further study process was given to participants in person (cohort) or via post after randomization (RCT).

In case of stroke, patients that could not be reached (e.g. due to access restrictions because of the COVID-19 pandemic) were contacted via letter (postal contact only from March 2022 onwards). Furthermore, information material was distributed via rehabilitation clinics, self-help organizations and other support offers, with contact details of the study coordination for patients that were interested in participating.

**Table 1:** Description of the selected sites for recruiting participants with stroke and lung cancer for the CoreNAVI study

Setting	Location	Recruiter	Estimated participant numbers^a^	Recruitment period
Stroke units (inpatient)	Berlin	Project study assistant	365 participants (215 RCT, 150 cohort)	June 2021– July 2022
Stroke unit (inpatient)	Brandenburg	Project study assistant	319 participants (244 RCT, 75 cohort)	January 2022– September 2022
Specialized lung cancer clinic (outpatient)	Berlin	Project study assistant	168 participants (98 RCT, 70 cohort)	August 2021– July 2022
Lung cancer hospital unit (inpatient)	Brandenburg	In-house personnel	22 participants (17 RCT, 5 cohort)	April 2022– September 2022

^a^Estimated numbers were calculated from hospitals’ patient numbers for the previous years with the following assumptions: 60-70% of patients can be screened as eligible for recruitment, and of these 30-50% of patients will take part in the RCT

For the cohort, we expected an additional participation rate of 30% of those that were not willing to participate in the RCT

### Screening documentation

The screening and recruitment process was documented in detail by the recruiters throughout the recruitment phase. All patients presenting to the recruitment sites during the recruitment period, irrespective of whether the inclusion criteria were fulfilled, were listed daily. Parameters documented included age, sex, co-morbidities (in Berlin stroke population), and stroke severity as modified Rankin Scale (mRS: the mRS is a standardized measure of disability following a stroke ranging from 0 [no symptoms] to 6 [death] [18, 19]) (in Berlin stroke population). Study enrolment was then documented. Participants were defined as patients that gave written informed consent to participate in either of the study arms (RCT or cohort). Reasons for non-participation were also documented. This included documentation of the predefined exclusion criteria (resident at a nursing home, residency outside of Berlin or Brandenburg, language barrier, cognitive impairment/unable to give informed consent) and other reasons (refused participation, patient not approached, patient not found, participation in another trial, patient discharged/transferred, other). Multiple reasons could be reported for individual cases. After the recruitment process was completed, all documented data from the screening were anonymized.

### Refuser assessment

The aim of the refuser assessment was the evaluation of the patients’ individual reasons for refusing participation in the study. These reasons were assessed for patients who fulfilled the inclusion criteria and had been approached by a recruiter, but who did not want to take part in the study. For this, a refuser questionnaire/checklist was prepared with predefined reasons (e.g. study not perceived as relevant, feeling overwhelmed, bad experience with studies, worries about data protection) and basic parameters (age, sex). Patients were asked to fill out the questionnaire themselves or with the help of study personnel. Alternatively, if the patient stated a reason for non-participation during the conversation with the recruiter, this was documented in the refuser checklist. Data was collected anonymously without any connection to names or case numbers.

### Analysis process

All data from the screening documentation and refuser assessments were collected in MS Excel databases throughout the recruitment process by the recruiters. Frequencies and percentages from the refuser assessments were calculated in MS Excel. Descriptive analyses of the data from the screening documentation (study participation, exclusion criteria, reasons for not approaching participants, age, sex, co-morbidities, disease severity) were calculated using IBM SPSS (Version 27). Flow charts of the recruitment process were prepared with frequencies for reasons for non-participation. The mean age with standard deviation, as well as absolute and relative frequencies for sex, disease severity (measured by mRS; only for stroke patients in Berlin) and selected co-morbidities (stroke patients in Berlin) were calculated.

### Qualitative interviews with recruiters

To evaluate the chosen recruitment strategy and to capture the recruiters’ experiences, we conducted qualitative interviews with the recruiters at regular intervals. A total of eight face-to-face interviews with four different recruiters (3 female, 1 male) in Berlin and Brandenburg were conducted in the recruiters’ offices between September 2021 and October 2022. The interviews with the same recruiters were conducted during and at the end of the recruitment process in order to capture their experiences and possible adjustments to the recruitment process. The interviews conducted after the recruitment phase aimed to obtain a final impression from the recruiters as to what they considered to be positive and negative factors for recruitment in relation to the chosen settings and timing.

The recruiters had either a professional background in nursing with experience in recruiting or in social work. The interviewer was an experienced qualitative researcher with Master’s degrees in communication sciences and public health. With regard to the interviewer-interviewee relationship, all participants were project staff and acquainted with each other. Interviews lasted between 30 and 80 min and were audio recorded and transcribed verbatim. An interview guide was developed based on an extensive literature review on experiences with recruitment strategies and the expertise of members of the study group, focusing on two main topics and research questions: (1) Are the chosen settings suitable for approaching potential study participants from the recruiters’ perspective? If yes, why? If no, why not? (2) Is the timing of the approach of the patients well chosen? If yes, why? If no, why not? (For the detailed interview guide, see Supplementary Information 1)

Thematic analysis was chosen as the methodological approach for analysing the qualitative data [20]. The guiding themes underlying the analysis were: (1) the settings for recruitment, (2) the timing of approaching patients, (3) challenges during the recruitment process, and (4) reasons for refusal for study participation. Accordingly, deductively defined categories for the thematic analysis of the interview data material were: *setting*,* timing*,* challenges*, and *refusal of study participation*. All relevant text passages were assigned to these categories. To ensure the quality of the research process and data analysis, the coding process was discussed regularly with the principal investigator (CH). 

Note that quantitative and qualitative results are analysed and presented separately in the results section.

## Results

### Screening and recruitment analysis

In the following section, the results of the screening and recruitment documentation are presented in detail, separately for stroke and lung cancer. For direct comparison of the characteristics of all screened patients with stroke and lung cancer, see also Supplementary Material 2.

#### Stroke

**Fig. 1:**
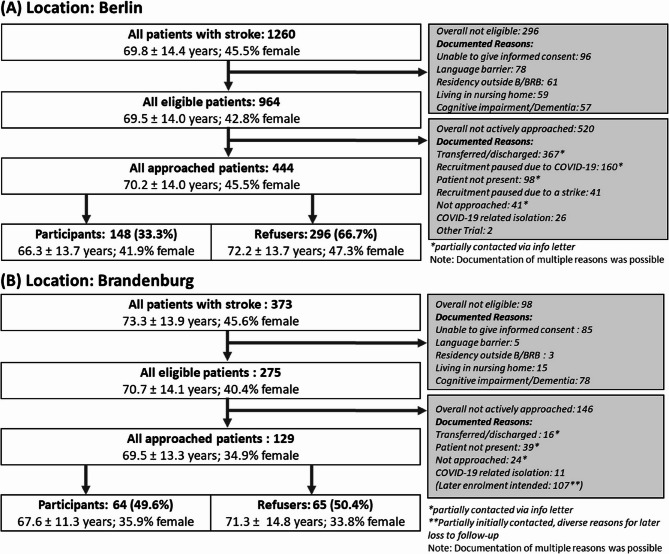
Flowcharts of recruitment processes for study participants with stroke in inpatient settings in the metropolitan area Berlin (**A**) and the rural area Brandenburg (**B**). Patients’ mean age ± standard deviation (in years) as well as the percentage of female patients is given. Note that in some cases more than one exclusion reason was documented. B = Berlin; BRB = Brandenburg

During the recruitment phase, a total of 1633 patients with stroke were screened in the participating recruitment locations (1260 patients in Berlin; 373 patients in Brandenburg) (see Fig. 1). Of these, 76.5% (964 of 1260) of patients in Berlin and 74.0% (275 of 373) of patients in Brandenburg were eligible for study participation. The most common reason for non-eligibility was inability to consent (due to stroke-related cognitive deficits). In the Berlin locations (metropolitan area), the inability to give informed consent due to a language barrier (insufficient German language proficiency for study information) was another main exclusion reason, while this reason was rarely reported in the more rural Brandenburg. Of the eligible patients with stroke, 46.1% (444 of 964) of patients in Berlin and 46.9% (129 of 275) of patients in Brandenburg could be actively approached for study recruitment to any of the study arms. The main reason for the inability to approach a patient for recruitment in Berlin was that patients had already been discharged from the hospital or had been transferred to non-recruiting departments. In Berlin, a substantial number of patients could also not be reached in person due to COVID-19-related factors (e.g. COVID-19-related restrictions, patients in isolation). However, these patients were contacted via postal invitations. Furthermore, a main reason for not approaching patients in Brandenburg as well as Berlin was that they were not present in the room at the time of recruitment (e.g. because of diagnostic or treatment procedures). COVID-19-related reasons for not approaching patients played a minor role in Brandenburg, presumably due to timing (recruitment started later, when major access restrictions due to COVID-19 had already been lifted).

Overall, 33.3% (148 of 444 patients) of approached patients in Berlin and 49.6% (64 of 129 patients) of approached patients in Brandenburg could be enrolled into the study. Of all patients that agreed to take part in the study, 178 gave written consent to take part in the RCT and 34 in the cohort. Compared to the overall screened population of stroke patients, participating patients were slightly younger (Berlin: mean 66.3 [SD 13.7] years versus 69.8 [14.4] years; Brandenburg: 67.6 [11.3] years versus 73.3 [13.9] years) and were less often female compared to non-participants (Berlin: 41.9% versus 45.5% female; Brandenburg: 35.9% versus 45.6% female). In case of stroke patients in the inpatient setting in Berlin, we were further able to compare participating patients to refusers regarding selected co-morbidities and disease severity (mRS) (see Table 2). Here, participants tended to be less severely affected (had less stroke severity as measured by mRS) compared to the overall population and to refusers.

**Table 2:** Analysis of frequencies of selected co-morbidities and disease severity (mRS) for patients with stroke screened and recruited in Berlin in aninpatient setting. Missing values were excluded from calculation and the percentage (cases/valid N) was reported for co-morbidities

	**All patients with stroke** **N=1260**	**All eligible patients ** **N=964**	**All approached patients ** **N=444**	**Participants** **N=148**	**Refusers ** **N=296 **
No. of co-morbidities below *Mean (95% CI)*	1.73 (1.65; 1.81)	1.75 (1.66; 1.83)	1.96 (1.82; 2,1)	1.91 (1.7; 2.13)	1.98 (1.8; 2.14)
*Hypertension*	68.3% (761/1114)	69.0% (589/854)	71.2% (297/417)	68.0% (100/147)	73.0% (197/270)
*Atrial fibrillation*	25.8% (287/1113)	24.3% (207/851)	22.7% (94/415)	18.4% (27/147)	25.0% (67/268)
*Coronary heart disease*	17.1% (190/1109)	18.1% (154/852)	16.2% (67/414)	13.6% (20/147)	17.6% (47/267)
*Diabetes*	24.1% (268/1114)	22.7% (194/853)	23.6% (98/416)	19.7% (29/147)	25.7% (69/269)
*Lipometabolic disorder*	34.4 % (381/1107)	36.5% (310/850)	45.2% (188/416)	51.0% (75/147)	42.0% (113/269)
*Former Stroke*	21.1 % (234/1108)	20.6% (175/850)	22.7% (94/414)	17.0% (25/147)	25.8% (69/267)
*Former TIA*	5.7% (63/1110)	6.4% (54/850)	7.2% (30/414)	4.8% (7/147)	8.6% (23/267)
Active Smoker	23.7 % (263/1108)	24.8% (210/847)	28.3% (117/413)	29.9% (44/147)	27.4% (73/269)
mRS *Median (25; 75%)*	2.0 (0;4)	2.0 (0;3)	2.0 (0.25; 3)	1.0 (0;3)	2.5 (1; 4)

#### Lung cancer

**Fig. 2:**
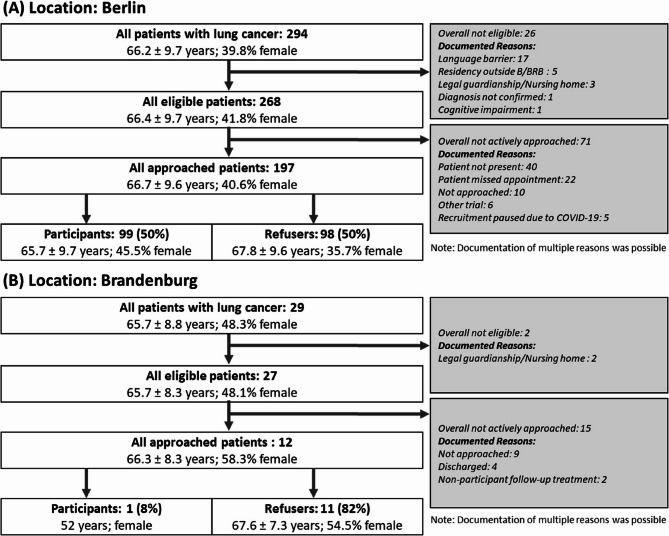
Flowchart of recruitment process for study participants with lung cancer in a specialized outpatient setting in the metropolitan area Berlin (**A**) and an inpatient setting in the rural area Brandenburg (**B**). Patients’ mean age ± standard deviation (in years) as well as the percentage of female patients are given. Note that in some cases, more than one exclusion reason was documented. B = Berlin; BRB = Brandenburg

A total of 323 lung cancer patients were screened (294 patients in Berlin; 29 patients in Brandenburg) (see Fig. 2). Of these, 91% (268 of 294) of patients in Berlin and 93% (27 of 29) of patients in Brandenburg were eligible to participate in the study. In Berlin, the main exclusion criterion was the inability to give informed consent due to a language barrier, while in Brandenburg two patients had to be excluded due to nursing home residency/having an existing legal guardianship at the time of recruitment. Of all eligible patients, 73.5% (197 of 268) in Berlin (inpatient setting) and 44% (12 of 27) in Brandenburg (outpatient setting) could be approached for recruitment. Of note, this latter percentage is consistent with the approach rate of 46% for the inpatient settings for stroke. While a key reason for not approaching patients in the inpatient setting was discharge before recruitment, for the lung cancer patients in the outpatient setting, most returned multiple times and thus there were more opportunities to approach them (number of visits per patient during the recruitment period [given as median, 25–75% percentile]: 5 visits [2–9]).

Of all approached patients, 50% (99 of 197 patients) agreed to take part in the study (RCT or cohort) in Berlin, while one patient took part from the Brandenburg recruitment site. Of all patients that consented to take part in the study, 68 gave written consent to take part in the RCT and 32 in the cohort. The age of participants in Berlin (mean: 65.7 ± 9.7 years) was comparable to the overall screened population of lung cancer patients in Berlin (mean: 66.2 ± 9.7 years).

### Assessment of reasons for refusing study participation

**Fig. 3:**
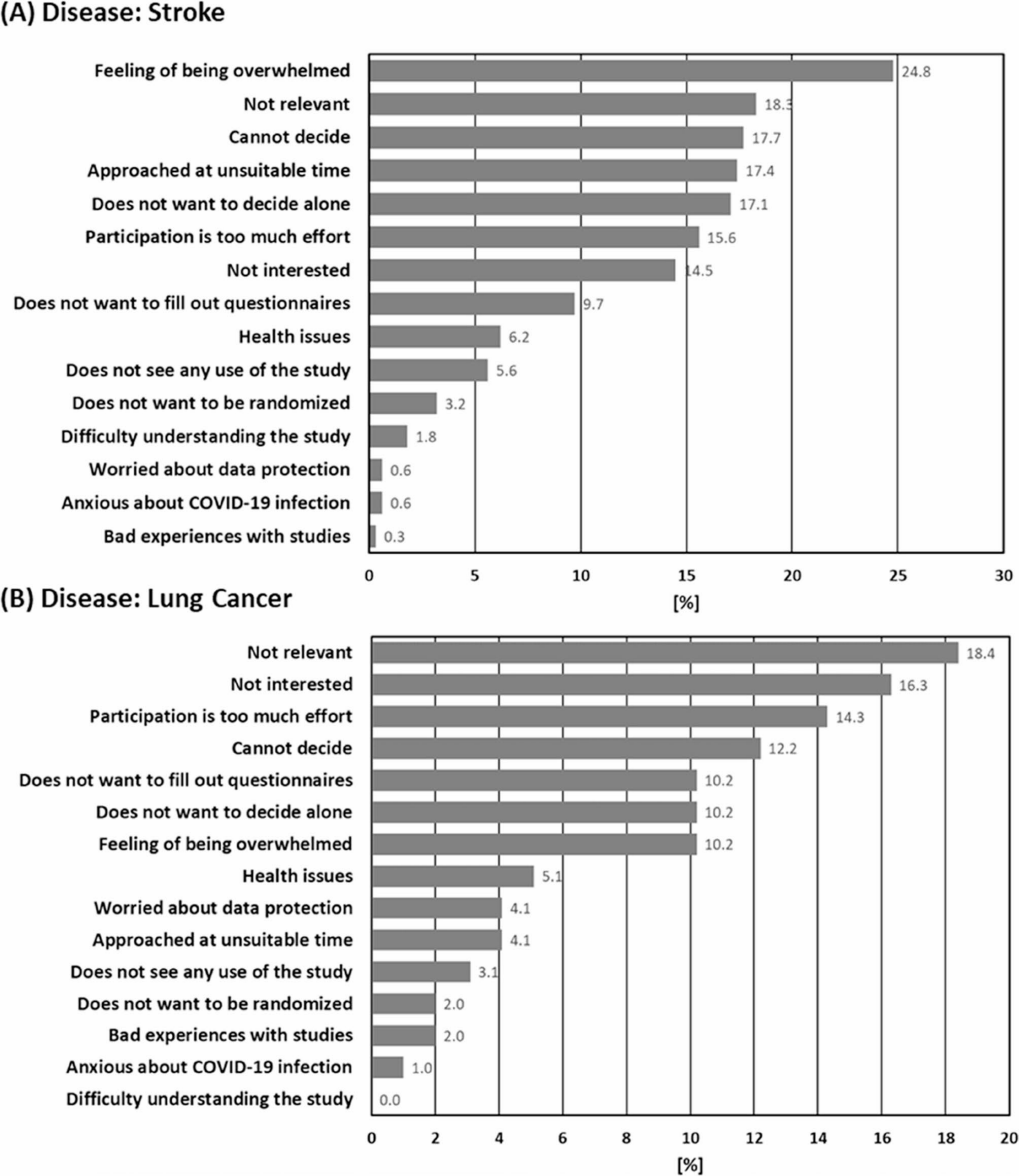
Reasons for refusing study participation for approached patients with stroke (**A**) and lung cancer (**B**), as documented by recruiters. In total, refuser assessments for 339 patients with stroke (271 patients in Berlin, 68 patients in Brandenburg) and 98 patients with lung cancer (86 patients in Berlin, 12 patients in Brandenburg) were analysed. The percentage of positive responses for all responses is calculated and shown for each item. Selection of multiple reasons was possible

Of the stroke patients approached for study recruitment, the most often documented reason for refusing participation was feeling overwhelmed (24.8% (84 of 339) patients). Perceiving the study as not relevant (18.3% (62 of 339) patients) and the contact for recruitment occurring at an unsuitable time of contact for recruitment (17.4% (59 of 339) patients) were other key reasons (see Fig. 3A). In the case of lung cancer patients, perceiving the study as not relevant (18.4% (18 of 98) patients) and generally not being interested (16.3% (16 of 98) patients) were the most frequently given reasons for refusing participation. Another key reason was the feeling that study participation would be too much effort (14.3% (14 of 98) patients) (see Fig. 3B). For all patients (stroke and lung cancer), difficulties to decide and the wish to not decide alone were also frequently documented reasons. Of note, for both patient groups, anxiety about a COVID-19 infection in the context of study participation was only a minor concern and negligible as a reason for refusing participation in the study (see Fig. 3A + B).

### Results of qualitative interviews with recruiters

In line with the thematic analysis of the interviews, results are presented here for the categories *setting*,* timing*,* challenges*, and *refusal of study participation*.

Interviews with the recruiters show that, from their perspective, the settings chosen to recruit stroke and lung cancer patients differed in terms of their suitability. Stroke patients were approached in the stroke unit, i.e. at a very early stage after the acute onset of the stroke event. From the recruiters’ perspective, patients’ mental overload and lack of cognitive capacity were the most important reasons why they could not be included in the study or refused to participate.

“So most often, I think, it’s simply being overwhelmed. Unfavourable timing, too many documents. Many people say that it’s really too much at that moment.” (Study Nurse_01_01).

“With stroke patients, it is often a bit difficult when they are very limited. If they can’t speak. They often can’t concentrate well; they can’t follow two sentences that you say to them.” (Study Nurse_01_02).

Another reason for refusing to participate in the study was that some patients felt well supported by their family and social networks and did not see any advantage in further support.

“And what also happens regularly is that many patients feel very well cared for. They already have other care support. They feel well taken care of.” (Study Nurse_01_02).

Reasons why stroke patients could not be approached were related to the organization of the ward and the treatment in the hospital: the daily routine was very tight and patients were busy with examinations or therapy and thus not available to recruiters. Furthermore, due to COVID-19, patients were very often transferred to other wards and also discharged from the stroke unit more quickly than usual.

“On the wards, of course, there is always the difficulty that when they go for examinations, they are often gone for hours. Patients who are mobile are no longer in their rooms in the afternoon. Then they wander around somewhere on the grounds. And sometimes it’s really difficult to see the patients and have the opportunity to conduct a recruitment interview in peace and quiet.” (Study Nurse_01_01).

“Then very high corona [COVID-19] numbers came again. Many patients with corona, who were then also positive in the normal ward. So, it felt as if the patients were transferred to another room every day, were discharged even faster, and especially in the inpatient area you notice that recruitment is really difficult because you simply don’t see many patients.” (Study Nurse_01_02).

In contrast, the lung cancer outpatient clinic setting was perceived as less challenging by the recruiters. The advantage here was, on the one hand, that many patients were on-site for several hours for treatment and thus the recruiters had sufficient time to approach them. The regularity of on-site treatment was also an advantage: if a patient could not be approached one week, it was usually possible at their next treatment appointment. As a result, a high number of all patients treated at the lung cancer outpatient clinic could be approached about possible study participation.

“So, in the lung tumour outpatient clinic, the challenges are quite minimal, because these patients keep coming back. They actually come, if they are in the therapy phase, at least once a week, so you can really approach them very well. And that is quite uncomplicated.” (Study Nurse_01_02).

However, the turbulent environment in the outpatient clinic was challenging. For example, patients were informed about the study during their chemotherapy treatment, during which there were frequent interruptions by the nursing staff doing their work.

“And sometimes you feel rushed and still want to do the procedure thoroughly. But it is too turbulent to be able to do it with a clear conscience. I am very OFTEN interrupted by nurses and doctors. That is understandable that they come, of course, but one always starts again. It is very uncomfortable to be interrupted every time. With patients, it was sometimes five or six times.” (Study Nurse_02_02).

Like the stroke patients, patients with lung cancer also stated as a reason for refusal that study participation would imply too much effort and paperwork or that they were already sufficiently well supported by their personal network.

“Many patients say it’s too much paperwork for them, too much effort. The vast majority say they have no need for support. Or they have a network that provides a lot of support.” (Study Nurse_02_01).

In summary, the qualitative results show that from the recruiters’ perspective, the acute stroke setting was significantly more challenging than the lung cancer outpatient setting. Recruiters recommended that rehabilitation clinics also be considered as possible recruitment settings for stroke patients. This would be a useful addition to the acute setting, also with regard to approaching potential study participants at a later point in their disease pathway.

“But I still think it was interesting to see this difference between tumor patients and these acute stroke patients. I think that is a very important experience. Because you can see it very well there, who are so completely taken by surprise by this acute, terrible event. I think they [stroke patients] are more difficult to approach than patients who have a diagnosis that shortens their lives. These [lung cancer patients] are already more in this situation, so it feels like we have better access to them. In addition, for the acute patients– it does not matter whether it is a stroke or a heart attack– it’s just this extreme surprise that is a bit more difficult. Perhaps we should wait during the acute phase and then address it at a later point in time, when, as I said, they have an overview of their condition. That is my impression.” (Study Nurse_01_03).

## Discussion

In this paper, we provide data on experiences gained and challenges encountered during patient recruitment into the CoreNAVI study, a feasibility study to investigate a patient navigation program for patients with stroke and lung cancer [17]. We showed detailed data on the screening and recruitment process; with final success rates for study enrolment from 8% of approached lung cancer patients in rural Brandenburg to 50% of approached lung cancer patients in Berlin and of approached stroke patients in Brandenburg. Notably, enrolment rate depended strongly on recruitment setting. We reported major organizational and context reasons for not reaching and not approaching patients at the recruitment sites. We further presented patients’ reasons for study refusal including feeling overwhelmed by the situation (stroke) and that the study is not perceived as relevant at the time of approach by the recruiter (lung cancer). These reasons were further analysed in a thematic analysis of the interviews with recruiters. Taken together, the initial assumptions of the rate of eligibility (60–70%) and recruitment rate (30–50%) were comparable with our observations. However, only 36% of initially estimated number of participants signed the consent form for study participation (with rates differing between settings, diseases and study arms). This was due to a high number of eligible patients that could not be actively approached for recruitment as well as due to shorter recruitment phases in some settings for organisational and personnel reasons.

As described in a previous review, reaching the pre-planned sample size for an RCT (but also for other observational studies) is a major challenge and is often not achieved [3–5]. In this present study, we were also unable to enrol the initially estimated number of participants (which was mainly based on the previously described assumptions: patient numbers from previous years, etc.). Based on our experiences and observations, we found that an overestimation of the number of eligible patients who could be directly approached was a major reason for low recruitment; a factor that was also described in a previous meta-analysis of barriers and enablers to patient recruitment [6].

We also propose that ‘real-life factors’ and unexpected events should be more thoroughly accounted for when planning recruitment for a trial. These may be organizational, contextual or recruiter-related. Recruitment usually takes place within an ongoing clinical work environment, where treatment and diagnostics obviously take priority over study recruitment. Due to relatively short hospital stays, this can effectively lead to a decrease in approached or available patients, especially if the recruiter is not in-house staff. We propose that further organizational reasons include changes and translocation of contact persons at the recruitment sites, as well as a setting in a university hospital where the number of competing studies and rotational personnel is high. In terms of the contextual factors that influenced our recruitment rates, unexpected events occurred that led to pauses in recruitment, including a labour strike and the COVID-19 pandemic. Finally, recruiter-related events included, for instance, a delay in hiring recruitment personnel due to personnel shortages, which led to a belated recruitment onset and hence an overall shorter recruitment period at some recruitment sites, as well as unexpected sick leaves. Accordingly, we conclude that these ‘real-world’ events should be adequately taken into account when planning future study recruitment, in order to more realistically plan for an achievable sample size.

In general, our results suggest that different trade-offs have to be taken into account in order to find an effective recruitment strategy. For our navigation program, we recruited stroke patients in the stroke unit and hence shortly after the acute stroke event. The rationale was to recruit in a setting where the population of stroke patients could be reached comprehensively; about 77% of all stroke patients are treated in specialized stroke units in Germany [21]. However, as evidenced by the results of our refuser assessment as well as the qualitative interviews with recruiters, this moment in a stroke patient’s disease trajectory may be too early, as they often feel overwhelmed by their new situation and will thus be unreceptive to the study invitation and the (complex) enrolment procedure. These factors have been discussed in previous studies on recruitment of patients with a variety of diseases [7, 12]. In addition, for our patient navigation intervention, these stroke patients may not (yet) have had the feeling of needing (additional) support, as they had not yet returned home to experience the changes of daily life due to the effects of the stroke.

On the other hand, in the case of the lung cancer patients we recruited in the specialized outpatient setting, here the advantage for recruitment was that patients had regular appointments and thus we were able to reach a high number of eligible patients. However, for some of the approached patients, the time point for recruitment might have been too late, as they were already at a later stage of an integrated care trajectory. This was indicated by the documented reason for participation refusal that the study was not perceived as relevant.

Another trade-off that must be decided upon during study planning is whether the recruitment staff should be in-house personnel or external project staff. In our case, we mainly recruited via the project’s study nurses, who were external to the participating departments. This had the advantage that study recruitment was their main focus and they did not have to balance different responsibilities. On the other hand, being external also poses some considerable challenges. This includes limited access to patient information, limited time spent at the recruitment site, less knowledge of the general organization of the department and the responsible personnel, as well as limited access to infrastructure like PCs, printers, etc. Setting up recruitment with external recruiters may thus require their integration into the team of the recruitment department, as well as the development of an effective strategy and workflow, which may prolong the run-in time of the recruitment phase until optimal recruitment numbers can be achieved.

On the other hand, with in-house personnel, difficulties may occur in terms of balancing their everyday care work in the hospital/outpatient clinic with their recruitment responsibilities. These recruitment responsibilities can be time consuming, as they include giving oral and written information to patients and caregivers, answering questions, giving support with filling out consent forms and, in our case, with the baseline assessment. We conclude, in accord with a previous study investigating the recruitment procedure of stroke patients into an intervention study [12], that dedicated in-house personnel should engage in recruitment as a primary responsibility. However, having a recruiter confined to one recruitment site and appointing full-time recruiters at participating sites may itself pose a challenge.

Finally, the balance between achieving the intended sample size and effectively recruiting the target population should be considered carefully at the outset [22]. As shown in our data, participating patients were slightly younger and tended to have a lower disease severity in case of stroke. For the recruitment of stroke patients, the bias towards lower disease severity has been described elsewhere, due to the inability of a high proportion of stroke patients to give informed consent [8, 9]. In addition, a limitation was that we had to exclude patients with a complete language barrier, as the project’s resources did not allow for the offer of translation during recruitment, and more importantly, the personal navigation intervention. This highlights another noteworthy point, however, that the complexity of the German healthcare system and the organization of personal care within this system may prevent patients with a migration history and a language barrier from optimally accessing available healthcare resources [23]; while such barriers may be overcome through effective navigation support. We aimed to circumvent the problem of excluding patients with cognitive deficits and full language barriers by opening up the opportunity for caregivers to participate in the study (either with the patient’s agreement or if legal guardianship existed). However, very few caregivers took up this opportunity. A major reason for this may be that recruitment took place during the COVID-19 pandemic when many restrictions were in place, and thus caregivers were either not allowed to visit patients or only for a very limited time.

The study has several limitations, on the one hand related to the recruitment period, which occurred during a time when hospital access restrictions due to the COVID-19 pandemic were still in place in varying extent. It should also be noted that the qualitative assessments and results about study participation and refusal might reflect the recruiters’ interpretations of the patients’ experiences rather than the patients’ perspectives themselves. While patient interviews were conducted as part of the underlying feasibility study, their primary focus was on participants’ experiences with the navigation process and support. Further, it should be underlined that the analysis of the documentation of the screening and recruitment process was conducted with the database used by recruiting staff as a working document during the recruitment process. Hence, slight differences in the documentation may appear due to the individual workflow of the recruiters, e.g. the reason ‘Later enrolment intended’ was used for the recruitment at Brandenburg site to document a variety of reasons like treatment/diagnostics at time of attempt approach, ongoing diagnostic to verify stroke diagnosis or an initial patient contact with an intended later contact that was lost to follow-up. These limitations should be kept in mind regarding the comparability and generalizability of the presented results.

## Conclusion

From our experience of recruiting patients for a study of a navigation intervention, we summarize the following lessons learned. First, we found that a recruitment strategy of actively approaching potential participants was effective. However, it should be broadened to include additional entry points along a patient’s care pathway, like rehabilitation clinics or outpatient practices. This would ensure not only reaching the target population comprehensively, but also reaching patients at a time when they are in need and receptive to the support offer; this can vary between individual patients, as well as between care pathways for different age-associated diseases. Second, the enrolment and initial study procedure should be as low-threshold as possible and include caregivers to avoid excluding the most severely affected patients and discouraging potential participants. This may include ideas such as the patient-oriented development of concise study information, which may additionally be provided in easy language, or providing short versions of baseline assessments. Finally, the potential occurrence and interference of real-world events during the recruitment phase should be factored in to a higher degree when planning the recruitment. Consideration of these organizational, contextual or recruiter-related factors might have an influence on recruitment endeavours in light of future developments of and challenges within the German healthcare system, such as personnel shortages and the decreasing lengths of hospital stays.

## Supplementary Information

Supplementary Material 1.

Supplementary Material 2.

Supplementary Material 3.

## Data Availability

The data are not publicly available for privacy and ethical reasons, but could be available from the corresponding author upon reasonable request.

## Abbreviations

RCT: Randomized controlled trial
TIA: Transient ischemic attack
mRS: Modified Rankin Scale
B: Berlin
BRB: Brandenburg
