# Supplementary material for: Recruiting patients into a healthcare services trial: lessons learned from a feasibility study to investigate a patient-oriented navigation intervention for age-associated diseases
Source: BMC Health Serv Res. 2025 Jul 2;25:883. doi: 10.1186/s12913-025-13023-x (PMC12224617; doi:10.1186/s12913-025-13023-x)
Supplement: Supplementary file 1 — Supplementary Material 1. [file 12913_2025_13023_MOESM1_ESM.docx]

**Supplementary information 1: Interview guide for interviews with recruiters**

| **Topic of *recruitment*** |
| --- |
| **Introductory question for the interview:**  *Please tell me about your experience as a recruiter. What is working well, what is not working so well from your point of view?*  **Questions about setting, timing, and challenges of recruitment:**   - *Please describe the settings for the recruitment of patients with stroke and lung cancer.* - *What are the challenges for the recruitment of stroke (or lung cancer) patients in the chosen setting?* - *What factors were supportive for your work as a recruiter?* - *Are there differences in the recruitment of stroke and lung cancer patients? If yes, what kind of differences?* - *In your opinion, what would be the best time to approach stroke patients (or lung cancer patients) for study participation?* - *Based on your experience, how would you assess setting and timing of the chosen recruitment strategy of stroke (or lung cancer) patients?* - *What would an ideal recruitment setting look like for you?* - *Why did patients refuse to participate in the study?* |
