# Supplementary material for: Recruiting patients into a healthcare services trial: lessons learned from a feasibility study to investigate a patient-oriented navigation intervention for age-associated diseases
Source: BMC Health Serv Res. 2025 Jul 2;25:883. doi: 10.1186/s12913-025-13023-x (PMC12224617; doi:10.1186/s12913-025-13023-x)
Supplement: Supplementary file 2 — Supplementary Material 2. [file 12913_2025_13023_MOESM2_ESM.pdf]

Supplementary table 1: Description of patient characteristics of all screened patients with stroke and lung cancer. Note that more that one exclusion reason could be documented.

		Stroke	Lung cancer
**Number of all screened patients**		1633	323
**Age in years (Mean ± SD)**		70.6 ± 14.4	66.1 ± 9.6
**Sex (% female)**		45.5	40.6
**Recruitment location N (%)**			
	Berlin	1260 (77.2)	294 (91)
	Brandenburg	373 (22.8)	29 (9)
**Reasons for non-eligibility N (%)**			
	Not able to give informed consent	181 (11.1)	-
	Cognitive impairment/Dementia	135 (8.3)	1 (0.3)
	Residency in Nursing home	74 (4.5)	5 (1.5)
	Residency outside Berlin/Brandenburg area	64 (3.9)	5 (1.5)
	Language barrier	83 (5.1)	17 (5.3)
